# The Winnipeg Intraspinal Pressure Monitoring Study (WISP): A protocol for validation of fiberoptic pressure monitoring for acute traumatic spinal cord injury

**DOI:** 10.1371/journal.pone.0263499

**Published:** 2022-09-20

**Authors:** Perry Dhaliwal, Marshall Wilkinson, Frederick A. Zeiler

**Affiliations:** 1 Department of Surgery, Section of Neurosurgery, Rady Faculty of Health Sciences, Winnipeg, Canada; 2 Health Sciences Centre, Shared Health Manitoba, Winnipeg, Canada; 3 Department of Human Anatomy and Cell Science, Rady Faculty of Health Sciences, Winnipeg, Canada; 4 Division of Anaesthesia, Department of Medicine, University of Cambridge, Cambridge, United Kingdom; 5 Biomedical Engineering, Faculty of Engineering, Winnipeg, Canada; 6 Centre on Aging, University of Manitoba, Winnipeg, Canada; ICORD, CANADA

## Abstract

**Background:**

Research efforts have been focused on limiting secondary injury after traumatic spinal cord injury by performing spinal decompression and early optimization of spinal cord perfusion. The Winnipeg Intraspinal Pressure Monitoring Study (WISP) was designed to validate the technique of intraspinal pressure monitoring at the site of injury using a fiberoptic pressure monitor placed at the site of injury.

**Objectives:**

To describe the design of the WISP study.

**Study design:**

Descriptive.

**Methods:**

We explain the current limitations in the available scientific literature around the topic of blood pressure management for acute traumatic spinal cord injury and rational for the WISP study. Then, we describe the design of WISP including the patient selection criteria, study interventions, follow up schedules and outcome measurements. A multitude of future research avenues are also discussed.

**Results:**

The WISP study is a single center pilot study designed to validate the technique of intraspinal pressure monitoring following acute traumatic spinal cord injury. The study involves the measurement of intraspinal pressure from within the subarachnoid space at the site of injury to derive a number of physiological parameters including spinal cord perfusion pressure, spinal cord blood volume, measures of spinal cord compliance and vascular reactivity indices. Twenty eligible patients will be recruited and followed for a period of 12 months with visits scheduled for the first 5 days and 1, 3, 6, and 12 months following surgical intervention.

**Conclusions:**

The WISP study will provide the first attempt in North America at validation of intraspinal pressure monitoring with a fiberoptic pressure monitor at the site of injury. Successful validation will lead to future studies to define optimal spinal cord perfusion pressure, relationships of neural injury biomarkers and outcomes as well as epigenetic studies.

**Trial registration:**

This study has been registered at clinicaltrials.gov (registration# NCT04550117).

## Introduction

In North America, the incidence of traumatic spinal cord injury (tSCI) is estimated to be 40 per million people [1]. To date, clinical research efforts have been focused on limiting the degree of secondary injury by performing spinal decompression [2] and optimizing spinal cord perfusion early after the traumatic event.

Optimizing blood flow to the spinal cord has been an ongoing area of research for decades [3]. The objective of improving blood flow to the spinal cord is to reduce the area of ischemia around the site of injury thereby reducing axonal loss and necrosis [4]. Numerous animal studies have been conducted to evaluate the effects of blood flow manipulation on spinal cord function [5]. Historically, a variety of models were used to induce tSCI with significant heterogeneity in the mechanism of injury and outcome measurements [6].

Similarly, data in humans regarding manipulation of blood pressure in the management of tSCI has limitations. A prior systematic review performed at our institution [6] identified 2 prospective studies and 7 retrospective studies that analyzed the relationship between hypertensive therapy and functional outcome in tSCI. Amongst these studies, no clear relationship could be made between hypertensive therapy and functional outcomes in patients with tSCI. Despite the paucity of data, current North American guidelines suggest maintaining mean arterial pressure between 85–90mmHg for 5–7 days after initial injury [7].

Recently, researchers have described the use of an intraspinal pressure monitor to better define spinal cord perfusion pressure(SCPP) [8]. In their preliminary observational study, the authors described the placement of a fiberoptic pressure monitor placed into the subarachnoid space directly at the site of tSCI. Intraspinal pressure readings were then recorded above, below and at the site of injury [9]. The authors then defined the components of the intraspinal waveform and correlated these components to patients’ respiratory rate, heart rate and opening of the aortic valve [10]. Attempts at manipulating the intraspinal pressure through hyperventilation and osmotic agents were deemed unsuccessful though use of vasopressors seemed to improve SCPP. In other reports, the authors successfully characterized correlation coefficients defining the reserve capacity of the spinal cord and autoregulatory curves for SCPP [11]. In doing so, the authors were able to derive an optimal SCPP based on recordings directly from the site of injury. In whole, this work represents the most direct and complete evaluation of the relationship between mean arterial pressure, intraspinal pressure and SCPP in humans.

Despite the elegance and significance of this work, the methodology has not been validated in any other institution to date. The Winnipeg Intraspinal Pressure Monitoring Study (WISP) aims to be the first North American study to validate the methodology of intraspinal pressure monitoring at the site of injury.

## Materials and methods

This study has been reviewed and approved by the University of Manitoba Biomedical Research Ethics Board (Ethics# HS23117(B2019:075).

### Study objectives

The primary objective of the WISP study is to validate the methodology of invasive intraspinal pressure monitoring to derive parameters for optimal SCPP, spinal cord reserve capacity and spinal reactivity index following tSCI. The following specific aims will be achieved through the primary objective:

1. The temporal profile of intra-spinal pressure (ISP) and arterial blood pressure (ABP) over the course of the patient’s SICU stay will be characterized, assessing the time series behaviors of ISP and ABP, and by determining SCPP as: ABP–ISP.
2. Feasibility of intra-compartmental compensatory reserve will be characterized by evaluating the relationship between slow-wave vasogenic fluctuations in the Fast Fourier Transformed pulse amplitude of ISP (called sAMP) and ISP over time.
3. Spinal cord vascular reactivity will be characterized continuously using the relationship between slow-wave vasogenic fluctuations in ISP or sAMP, and either mean arterial pressure (MAP) or SCPP.

Secondary objectives of the WISP study will be to a) evaluate the safety of invasive ISP monitoring, b) prospectively evaluate the relationship between SCPP and functional outcomes in patients with tSCI and c) evaluate the relationship between SCPP, motor evoked potentials and functional outcomes after tSCI.

### Patient population

The WISP study will be a single center pilot study involving patients with acute traumatic tSCI with American Spinal Injury Association Score of A, B or C (ASIA A, B or C) admitted to the surgical intensive care unit at Health Sciences Centre in Winnipeg, Manitoba, Canada. Approximately 20 patients with acute traumatic spinal cord injury present to the institution on an annual basis. Given that this is a feasibility and validation study, we will select a convenience sample of 20 for enrollment in this study. Patients are eligible for the WISP study if they meet all of the following criteria:

Inclusion Criteria:

- patients with tSCI (ASIA A, B or C)
- patients requiring surgical intervention for spinal instability
- age between 18–70yrs

Exclusion Criteria:

- patients with central cord syndrome
- patients presenting to HSC >48hrs from time of tSCI
- patients undergoing surgical decompression and fixation for tSCI >48hrs from time of spinal cord injury.
- patients unable to communicate in English language
- pre-existing cognitive impairment
- penetrating tSCI
- pre-existing neurodegenerative disorder involving brain or spinal cord
- patients with concomitant injuries requiring emergent surgical intervention

### Patient recruitment, enrollment and retention

Appendix A in the supplementary materials lists the time frame for enrollment, intervention and assessments. Research assistants will be employed to continually scan patient lists in the intensive care unit to identify potential candidates for enrollment into the study. A standardized recruitment script will be used to inform patients about the rationale for the study, risks of participation and potential benefits. Informed consent will be obtained based on institutional ethics guidelines. Long term retention of patients in the study will be supported via dedicated clinic follow ups with patients. Patients will not be compensated for participation in this study.

Study patients may withdraw voluntarily from the study or the investigator may terminate the patient’s participation if the subject meets an exclusion criterion (either newly developed or previously not recognized) or a medical condition occurs that precludes furthur study participation. In situations where a patient is withdrawn from the study, complete adverse events data will be captured and stored in case of future need. We will then aim to extend the duration of the study until study enrollment is complete.

### Study interventions

**Fig 1:**
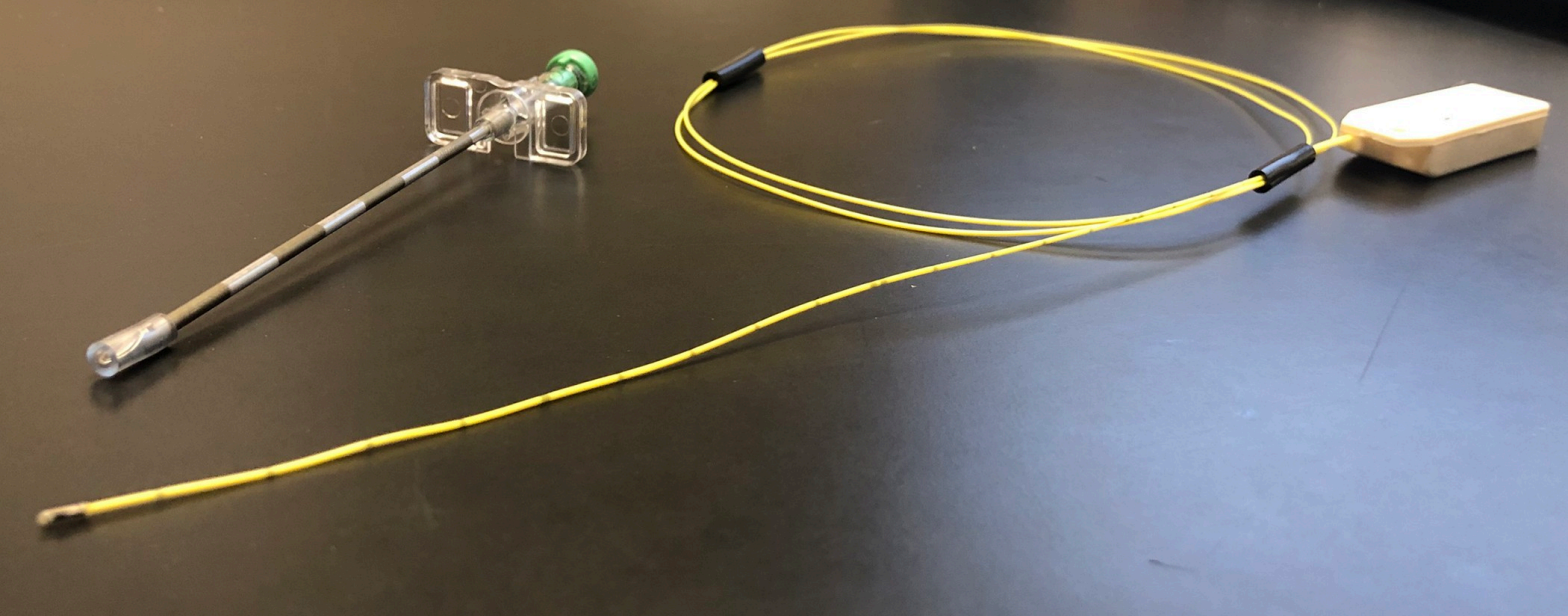
Fiberoptic monitor (Codman Microsensor Basic Kit, Integra LifeSciences Production Corporation, Mansfield, MA).

**Fig 2:**
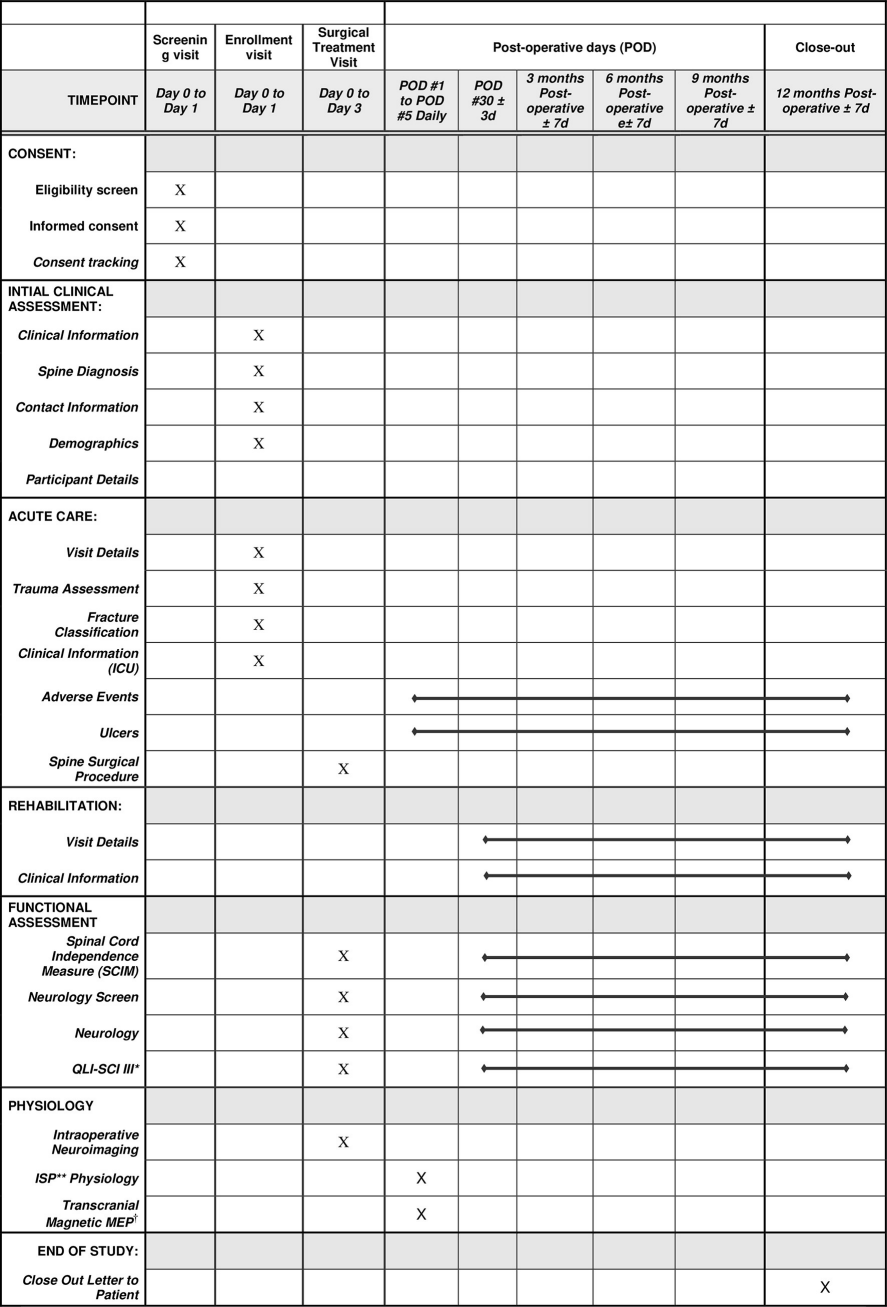
SPIRIT schedule of enrolment, interventions, and assessments. * QLI-SCI III: Quality of Life Index, Spinal Cord Injury Version III. ** Intraspinal Pressure. † Motor Evoked Potential.

Initial demographic information, medical history, physical examination and imaging findings will be recorded using standardized data collection forms for all patients. Patients will then be taken for surgical intervention within 24hrs of admission. A fiberoptic pressure monitor (Codman ICP MicroSensor; Integra LifeSciences Production Corporation, Mansfield, MA) will be placed into the subarachnoid space at the site of injury (Fig 1). The pressure monitor will be inserted by performing a laminectomy at the site of injury and one level above or below. A small durotomy will be made either rostral or caudal to the site of injury and the fiberoptic wire will be placed into the subarachnoid space. Care will be taken to ensure the fiberoptic wire is placed adjacent to but not within the spinal cord tissue under guidance with intraoperative ultrasound. The fiberoptic wire will then be sutured and secured to the skin with adhesive dressings. A small fat graft and fibrin glue will also be placed on the dura at the site where the fiberoptic cable enters the dura to prevent postoperative spinal fluid leak. The fiberoptic monitor will not be placed if a) there is a large dural defect from the traumatic injury that cannot be repaired primarily b) if there is significant subarachnoid hemorrhage preventing smooth entry of the monitoring device or c) if there severe spinal cord injury resulting in macerated or transected spinal cord tissue based on intraoperative assessment. Standardized data collection forms will be used to collect data related to the surgical intervention and any intraoperative adverse events. Patients will be admitted to the surgical intensive care unit for standard post-operative monitoring. Targets for mean arterial pressure will be determined by the treating physician and will not be pre-specified as part of this feasibility study. The fiberoptic wire will be removed at the bedside on the 6th post-operative day by removing the anchoring suture and sliding the monitor out of the surgical wound. The schedule of enrolment, interventions and assessments is detailed in Fig 2.

### Physiologic data capture and processing

During the care period in the SICU, intraspinal pressure and physiological parameters incuding heart rate, respiratory rate, blood pressure and mean arterial pressure will be captured at the bedside using a secure laptop for a maximum of 5 days following admission to the SICU.

Both ABP and ISP will be obtained through either radial or femoral arterial lines connected to pressure transducers (Baxter Healthcare Corp. CardioVascular Group, Irvine, CA), with all signals recorded in high frequency time series, sampled at 100 Hz or higher through and analogue to digital signal converters (DT9804 or 9T9826; Data Translations, Marlboro, MA), using ICM+ software (Cambridge Enterprise Ltd, Cambridge, UK, http://www.neurosurg.cam.ac.uk/icmplus) connected to our SICU monitors. Signals from all of the monitoring devices described below are subsequently recorded in time series using this software over the course of the recording periods described above. All physiologic signals from monitoring devices within the SICU for neurologically ill patients are currently recorded and archived as part of a separate prospective signal database study that will be ongoing at HSC.

Post-acquisition processing of the above signals will be conducted using ICM+ software. SCPP will be calculated using the formula: SCPP = MAP–ISP. Systolic ABP (ABPs) will be determined by calculating the maximum ABP over a 1.5 second window, updated every second. Similarly, diastolic ABP (ABPd) will be determined by calculating the minimum ABP over a 1.5 second window, updated every second. Pulse amplitude of ISP (sAMP) will be determined by calculating the fundamental Fourier amplitude of the ISP pulse waveforms over a 10 second window, updated every 10 seconds.

Ten second moving averages (updated every 10 seconds to avoid data overlap) will be calculated for all recorded signals: ISP, ABP (which produced MAP), ABPs and ABPd. Ten second moving averages will be calculated in order to focus on slow-waves of parent signals, decimating the frequency to the range associated with vascular autoregulation.

Spinal vascular reactivity indices(sPRx) will be derived as follows: a moving Pearson correlation coefficient will be calculated between ISP and MAP using 30 consecutive 10 second windows (ie. five minutes of data), updated every minute. Similarly, other vascular reactivity metrics will be derived using the correlations between slow-waves of sAMP and MAP (creating spinal pulse amplitude index; sPAx), and sAMP and SCPP (creating sRAC; R = correlation, A = pulse amplitude of ISP, C = SCPP). Finally, spinal compensatory reserve will be assessed by deriving the spinal RAP index (sRAP), using the moving Pearson correlation between sAMP and ISP. Data for further analysis will be provided in the form of a minute by minute time trends, output into comma separated values (CSV) datasets.

### Motor evoked potentials

Patients with incomplete tSCI (ASIA grade C) will undergo evaluation for motor evoked potential monitoring. Motor evoked potentials (MEP) will be acquired using single pulse transcranial magnetic stimulation (TMS) via a figure of 8 induction coil (Medtronic MagstimPro). Bilateral surface recordings from the thenar eminence will be attempted with targeted stimulation of the motor cortex hand area. The ground lead will be placed on the dorsal surface of the hand unilaterally. The MEP recordings will be amplified as required and bandpassed filtered (30 – 3000Hz). The stimulus location is typically 8 cm lateral to midline at a point 2 cm posterior to the coronal suture. 100% of coil output will be applied unless robust MEP are detected whereby the stimulus intensity can be titrated downwards. MEP peak-to-peak amplitude (μV) and onset latency (ms) will be registered. The MEP data will be captured and stored using a Cadwell Elite neuromonitoring workstation. Between 5–10 trials will be averaged if MEP responses are small (< 50 μV). Any changes in these MEP characteristics will be noted as arterial pressure optimization is achieved. We will capture transcranial magnetic motor evoked potentials through the first 5 days after surgery and during follow up assessments at 1, 3, 6, 9 and 12 months.

### Follow up

Post-operative adverse events will be collected for the first 30 days following surgical intervention. Thereafter, each patient will undergo standardized functional assessments at 1, 3, 6, 9 and 12 months in a dedicated multidisciplinary clinic involving spine surgeons, physiatrists, physical therapists and occupational therapists.

### Outcome measures

#### Primary outcome measures

The outcome measures for our primary objective will focus on various signal processing techniques to derive ISP, MAP, SCPP, sAMP, sRAP and various spinal cord vascular reactivity metrics during the post-acquisition processing phase.

#### Secondary outcome measures

Safety of intraspinal pressure monitoring will be measured based on accuracy of placement of the probe from post-operative computed tomography (CT) scan imaging, as well as collection of adverse events including infection rates, rates of pseudomeningoceles, neurological injury, probe dislodgement, meningitis, and/or subdural hematoma at site of probe placement.

Functional outcomes will be determined by examining changes to the total score on the ASIA impairment scale, Spinal Cord Independence Measure (SCIM III) and Quality of Life Index for Spinal Cord Injury (QLI-SCI) at the planned follow up intervals.

Changes in motor evoked potentials will be measured for patients with incomplete spinal cord injury with some preservation of motor function (ASIA C). Alterations in the MEP signals will be evaluated in relation to spinal cord perfusion parameters.

### Study management

#### Study oversight

The investigators will be responsible for study oversight to ensure that the study is conducted according to protocol and ensuring data integrity. Investigators will review the data for safety concerns at regular intervals, and will promptly report any unanticipated problems, protocol deviations, or any other significant event that arises during the conduct of the study to the research ethics board. A data safety monitoring committee will be convened as to ensure patient safety during this feasibility study.

#### Safety monitoring

All medical and surgical adverse events will be recorded on dedicated forms at the prescribed follow up intervals. A list of the adverse events that will be captured are included in Appendix B in the Supplementary Information. The severity of the adverse event will be categorized based on the need for minor or complex treatment, need for surgical intervention or ICU stay, anticipated duration or impact of the adverse event and threat to life. All serious adverse events will be reported to the research ethics board within 1 week of the investigator becoming aware of the event. Serious adverse events are defined as those that i) result in death, ii) are life threatening, iii) result in prolongation of existing hospitalization, or iv) result in persistent or significant disability. A safety monitoring committee will be convened to review all adverse events during the study. The safety monitoring committee will consist of the primary investigators, critical care physicians and neurosurgeons. The committee will review all adverse events for every cohort of 5 patients enrolled into the study. To ensure timely review of adverse events, the data safety monitoring committee will review adverse events within the first 30 days of the surgical and study interventions.

### Statistical analysis

#### General statistics

Statistical analysis will be performed utilizing R statistical software (R Core Team (2018). R: A language and environment for statistical computing. R Foundation for Statistical Computing, Vienna, Austria. URL https://www.R-project.org/). Alpha for statistical significance will be set at 0.05, with normality for all continuous variables tested via Shapiro-Wilks test. Basic descriptive statistics will be performed, with comparison between groups and variables conducted via t-test, Mann-Whitney-U, chi-square, analysis of variance (ANOVA), Kruskal-Wallis, Friedman and Joncheere-Terpstra testing, where appropriate. General correlations will be described using Pearson/Spearman coefficients, where applicable.

Descriptive statistical analysis of the relationships between physiologic variables will be facilitated through various error bar plots, linear models, quadratic models (i.e. For optimal SCPP determination) and locally weighted scatterplot smoothing (LOESS) techniques, depending on the physiologic relationship being assessed.

#### Time series techniques

All indices of cerebrovascular reactivity will be output in minute-by-minute time series format. Correlation between ISP and MAP slow-waves will be further evaluated in time series using cross correlation techniques, cross-correlation function (CCF) plots, vector autoregressive integrative moving average (VARIMA) models, impulse response function (IRF) plots and Granger causality testing. Further, using Box-Jenkins time series modelling, the autoregressive integrative moving average (ARIMA) structure of each time series will be assessed and compared. ARIMA model accuracy for each index will be confirmed using autocorrelation function (ACF), partial autocorrelation function (PACF) plot, augmented Dickey-Fuller (ADF) and Kwiatkowski–Phillips–Schmidt–Shin (KPSS) testing, and the presence of random normally distributed residuals. VARIMA and ARIMA model superiority will be confirmed via ANOVA testing and comparing Akaike information criterion (AIC), Bayesian information criterion (BIC) and log-likelihood (LL).

#### Association with secondary outcomes

ISP, MAP, SCPP, sAMP, sRAP and the spinal vascular reactivity indices will be evaluated in relationship to motor evoked potential recordings, and functional outcome measures at the defined time periods of assessment. Such analysis will involve linear and logistic regression modelling techniques, evaluating the relationship between the physiologic parameters with: ASIA, SCIMIII and QLI-SCI functional outcome metrics.

## Discussion

Blood pressure management after tSCI has been an area of interest for many decades. Researchers have attempted to define mechanisms of spinal cord autoregulation and perfusion that serve to protect the spinal cord penumbra around the site of injury.

The WISP study aims to validate the technique of intraspinal pressure monitoring at the site of injury. Other researchers [12, 13] have demonstrated a strong interest in assessing spinal cord perfusion through the use of lumbar drain catheters to measure cerebrospinal fluid dynamics. There is considerable equipoise in the consideration of these differing techniques. Direct intraspinal pressure measurement at the site of injury has been shown to better represent the parenchymal pressure and allows for direct derivation of autoregulatory curves [9]. Thus, validation of this technique will serve to clarify whether direct monitoring of intraspinal pressure at the site of injury provides more personalized information about the state of spinal cord perfusion for a patient.

To our knowledge, no other group has attempted to validate this technique since it was originally conceptualized. Through the WISP study, we expect to demonstrate the feasibility and safety of introducing a fiberoptic pressure monitoring probe in the subarachnoid space at the site of injury. We will be able to derive important relationships to assess spinal cord blood volume, measures of spinal cord compliance and vascular reactivity indices.

The WISP study may have significant implications for patients with tSCI. To date, broad based guidelines have recommended maintenance of the mean arterial pressure greater than 85mmHg. However, this overlooks the various metabolic factors that could alter spinal cord autoregulation. Continuous monitoring of intraspinal pressure at the site of injury holds the potential for individualized physiological targets and the possibility of preserving more functional spinal cord tissue around the site of injury.

Following successful completion of the WISP study, we anticipate several future avenues for research. First, there will be a need for future large multicenter network of patients being monitored for SCPP, aimed at providing multi-center validation of the technique and results. This will be facilitated through collaborative networks within Canada, US and Europe. As the understanding around optimal SCPP is still very much in its infancy, we expect numerous local and multi-center studies evaluating: feasibility of derivation, determinants of yield in calculation, baseline patient demographic associates, and the link to long-term functional outcome after tSCI. Finally, aside from exploring novel personalized physiologic targeting in tSCI, there is also the need to link this high-fidelity intra-spinal pressure data with other omics data. We envision such omics data to encompass genomic, epigenomic and proteomic information, integrated with high-frequency physiome from intraspinal monitoring and derived metrics. Future work at our lab is planned to evaluate the association between cerebrospinal fluid and serum proteome and small molecule profile for markers of neural injury, pro-inflammatory cytokines and vascular mediators. As in the traumatic brain injury literature, it is expected that individual genetic variation, in the form of single nucleotide polymorphisms, may dictate the host response to injury, both systemically and within the central nervous system.

## Supporting information

S1 Checklist (DOC)

S1 Appendix Minor and major adverse events list. (DOCX)

S2 Appendix Study protocol. (DOC)
