# Supplementary material for: The Winnipeg Intraspinal Pressure Monitoring Study (WISP): A protocol for validation of fiberoptic pressure monitoring for acute traumatic spinal cord injury
Source: PLoS One. 2022 Sep 20;17(9):e0263499. doi: 10.1371/journal.pone.0263499 (PMC9488753; doi:10.1371/journal.pone.0263499)
Supplement: S1 Appendix — (DOCX) [file pone.0263499.s002.docx]

| **Appendix B - Minor and Major Adverse Events List**  **Medical Adverse Events**  Arrhythmia |
| --- |
| Asthma/COPD exacerbation |
| Angina (unstable or new onset) |
| Bowel ileus |
| Bowel obstruction |
| Cerebrovascular event |
| Coagulopathy |
| CHF (new onset) |
| DVT |
| Delirium |
| GI bleed |
| MI |
| Cardiac arrest |
| Pressure point - skin |
| Pressure point - peripheral nerve |
| Pneumonia |
| Pulmonary embolus |
| Sepsis |
| UTI |
| Other |
| **Surgical Adverse Events** |
| Bone graft harvest site |
| CSF leak post op |
| Graft dislodgement or failure |
| Hardware Loosening or failure |
| Hardware misplacement |
| Hematoma |
| New onset pain |
| Neurological Deterioration |
| Cord injury with > 1 motor grade deterioration on ASIA scale |
| Nerve root weakness >1 MRC grade |
| Cauda equina syndrome |
| Wound Infection (superficial vs deep) |
| Wound dehiscence  Other |
