# Supplementary material for: The Winnipeg Intraspinal Pressure Monitoring Study (WISP): A protocol for validation of fiberoptic pressure monitoring for acute traumatic spinal cord injury
Source: PLoS One. 2022 Sep 20;17(9):e0263499. doi: 10.1371/journal.pone.0263499 (PMC9488753; doi:10.1371/journal.pone.0263499)
Supplement: S2 Appendix — (DOC) [file pone.0263499.s003.doc]

# Intraspinal Pressure Monitoring for Acute Traumatic Spinal Cord Injury: A Validation Study

Primary Investigator: Perry Dhaliwal, MD MPH

Co-Investigator: Frederick A. Zeiler, MD PhD

Version 3.0

November 09, 2021

Study Site: Health Sciences Centre

Contact Information:

Perry Dhaliwal, MD MPH FRCSC

Assistant Professor, Section of Neurosurgery

University of Manitoba

GB 127C - 820 Sherbrook Street

Winnipeg, Manitoba

R3A 1R9

Email: [pdhaliwal@exchange.hsc.mb.ca](mailto:pdhaliwal@exchange.hsc.mb.ca)

Telephone: 204-807-6728

Fax: 204-783-7356

# Table of Contents

[**1. Key Personnel** 6](#__RefHeading___Toc14382144)

[*2. Introduction: Background and Rationale* 7](#__RefHeading___Toc14382145)

[*3.* *Objectives* 8](#__RefHeading___Toc14382146)

[3.1 Study Objectives 8](#__RefHeading___Toc14382147)

[3.2 Study Outcome Measures 9](#__RefHeading___Toc14382148)

[*4. Study Design* 10](#__RefHeading___Toc14382151)

[4.1 Patient Population 10](#__RefHeading___Toc14382152)

[4.2 Physiologic Data Capture and Processing 10](#__RefHeading___Toc14382153)

[4.3 Motor Evoked Potentials 11](#__RefHeading___Toc14382154)

[5.2 Exclusion Criteria 12](#__RefHeading___Toc14382155)

[5.3 Strategies for Recruitment and Retention 12](#__RefHeading___Toc14382156)

[5.4 Subject Withdrawal 12](#__RefHeading___Toc14382157)

[5.5 Premature Termination or Suspension of the Study 12](#__RefHeading___Toc14382158)

[*6. Study Schedule* 13](#__RefHeading___Toc14382159)

[6.1 Schedule of Evaluations 13](#__RefHeading___Toc14382160)

[6.2 Description of Evaluations 13](#__RefHeading___Toc14382161)

[*7. Safety Assessments* 15](#__RefHeading___Toc14382168)

[7.1 Unanticipated Problems 15](#__RefHeading___Toc14382169)

[7.2 Serious Adverse Events 15](#__RefHeading___Toc14382170)

[7.3 Reporting Procedures 16](#__RefHeading___Toc14382171)

[7.4 Study Oversight 16](#__RefHeading___Toc14382172)

[*8. Statistical Considerations* 16](#__RefHeading___Toc14382173)

[8.1 General Statistics 16](#__RefHeading___Toc14382174)

[8.2 Time Series Techniques 17](#__RefHeading___Toc14382175)

[8.3 Association with Secondary Outcomes 17](#__RefHeading___Toc14382176)

[*9. Source Documents and Access to Source Data/Documents* 17](#__RefHeading___Toc14382177)

[9.1 Data Handling and Record Keeping 18](#__RefHeading___Toc14382178)

[9.2 Data Management Responsibilities 18](#__RefHeading___Toc14382179)

[*10 Ethics And Protection of Human Subjects* 18](#__RefHeading___Toc14382180)

[10.1 Research Ethics Board Submissions 18](#__RefHeading___Toc14382181)

[10.2 Informed Consent 18](#__RefHeading___Toc14382182)

[10.3 Patient Confidentiality 19](#__RefHeading___Toc14382183)

[11. References 19](#__RefHeading___Toc14382184)

# Abbreviations

| ABP | Arterial blood pressure |
| --- | --- |
| ABPd | Diastolic arterial blood pressure |
| AIC | Akaike information criterion |
| ADF | Augmente Dickey-Fuller |
| ANOVA | Analysis of variance |
| ARIMA | Autoregressive integrative moving average |
| ASIA | American Spinal Injury Association |
| CCF | Cross Correlation Function |
| CRF | Case report form |
| CT | Computed tomography |
| FIM | Functional Independence Score |
| HSC | Health Sciences Center |
| IRF | Impulse response function |
| KPSS | Kwiatkowski–Phillips–Schmidt–Shin |
| LL | Log likelihood |
| SICU | Surgical Intensive Care Unit |
| ISP | Intraspinal pressure |
| MAP | Mean arterial pressure |
| PI | Principal Investigator |
| POD | Post-operative day |
| QLI-SCI | Quality of Life Index for Spinal Cord Injury |
| RAP | Cerebral compensatory reserve index |
| REB | Research ethics board |
| SAE | Serious Adverse Events |
| sAMP | Pulse amplitude of intraspinal pressure |
| sPAx | Spinal pulse amplitude index |
| sPRx | Spinal pressure reactivity index |
| sRAP | Intra-spinal compensatory reserve |
| SCPP | Spinal cord perfusion pressure |
| SLICS | Subaxial Cervical Spine Injury Classification and Severity Scale |
| TLICS | Thoracolumbar Injury Classification Score |
| VARIMA | Vector autoregressive integrative moving average |

# Protocol Summary

| Title | Intraspinal Pressure Monitoring for Acute Traumatic Spinal Cord Injury: A Validation Study |
| --- | --- |
| Objectives | The primary objective of this study is to validate the methodology of invasive intraspinal pressure monitoring to derive parameters for optimal spinal cord perfusion pressure, spinal cord reserve capacity and spinal reactivity index using data obtained during the patient’s SICU stay.Secondary objectives of this study will be to a) evaluate the safety of invasive intraspinal pressure monitoring, b) prospectively evaluate the overall relationship between spinal cord perfusion pressure and functional outcomes in patients with acute traumatic spinal cord injury and c) evaluate the relationship between spinal cord perfusion pressure, motor evoked potentials and functional outcomes after *incomplete* spinal cord injury. |
| Population | Adult (age 18-75) patients admitted with ASIA grade A, B, or C acute traumatic spinal cord injury |
| Number of Sites | Health Sciences Center |
| Study Duration | 3 years |
| Subject Participation Duration | 1 year |
| Estimated Time to Complete Enrollment | 2yrs |

# Key Personnel

**Primary Investigator:** Perry Dhaliwal, MD MPH

**Co-Investigator:** Frederick A. Zeiler, MD PhD

**Study Coordinator:** Arsalan Alizadeh MD PhD

GF-241 Health Sciences Centre

820 Sherbrook St.

Winnipeg, MB

R3A 1R9

Phone: (204) 295-3264

## Introduction: Background and Rationale

Acute traumatic spinal cord injury has devastating consequences for patients and their families. In North America, the incidence of acute traumatic spinal cord injury is estimated to be 40 per million people^1^. To date, clinical research efforts have been focused on limiting the degree of secondary injury by performing spinal decompression^2^ and optimizing spinal cord perfusion early after the traumatic event.

Optimizing blood flow to the spinal cord has been an ongoing area of research for decades^3^. The objective of improving blood flow to the spinal cord is to reduce the area of ischemia around the site of injury thereby reducing axonal loss and necrosis^4^. Numerous animal studies have been conducted to evaluate the effects of blood flow manipulation on spinal cord function^5^. Historically, a variety of models were used to induce spinal cord injury with significant heterogeneity in the mechanism of injury. Similarly, significant heterogeneity exists with respect to measurement of outcomes where some studies observed changes in spinal cord blood flow whereas others have documented changes in neurological function^6^. However, these studies have several limitations. Many of these studies manipulated blood pressure over short periods of time and similarly evaluated functional outcomes within a very short time period following the spinal cord injury.

Similarly, data in humans regarding manipulation of blood pressure in the management of spinal cord injury is limited. A prior systematic review performed at our institution^6^ identified 2 prospective studies and 7 retrospective studies that analyzed the relationship between hypertensive therapy and functional outcome in acute traumatic spinal cord injury. Amongst these studies, no clear relationship could be made between hypertensive therapy and functional outcomes in patients with traumatic spinal cord injury. Despite the paucity of data, current North American guidelines suggest maintaining mean arterial pressure between 85–90mmHg for 5–7 days after initial injury^7^.

Recently, researchers at the University of Cambridge have described the use of an intraspinal pressure monitor to better define spinal cord perfusion pressure^8^. In their preliminary observational study, the authors described the placement of a fiberoptic pressure monitor placed into the subarachnoid space directly at the site of spinal cord injury. Intraspinal pressure readings were then recorded above, below and at the site of injury. The authors then defined the components of the intraspinal waveform and correlated these components to patients’ respiratory rate, heart rate and opening of the aortic valve. Attempts at manipulating the intraspinal pressure through hyperventilation and osmotic agents were deemed unsuccessful though use of vasopressors seemed to improve spinal cord perfusion pressure. In other reports, the authors successfully characterized correlation coefficients defining the reserve capacity of the spinal cord and autoregulatory curves for spinal cord perfusion pressure. In doing so, the authors were able to derive an optimal spinal cord perfusion pressure based on recordings directly from the site of injury. In whole, this work represents the most direct and complete evaluation of the relationship between mean arterial pressure, intraspinal pressure and spinal cord perfusion pressure in humans.

Despite the elegance and significance of this work, the methodology has not been validated in any other institution to date. We aim to be the first group to validate the methodology of intraspinal pressure monitoring.

## Objectives

### ***Study Objectives***

The primary objective of this study is to validate the methodology of invasive intraspinal pressure monitoring to derive parameters for optimal spinal cord perfusion pressure, spinal cord reserve capacity and spinal reactivity index using data obtained during the patient’s SICU stay (or a maximum of 5 days after injury). The following specific aims will be achieved through the primary objective:

1. The temporal profile of intra-spinal pressure (ISP) and arterial blood pressure (ABP) over the course of the patient’s SICU stay will be characterized, assessing the time series behaviors of ISP and ABP, and by determining the spinal cord perfusion pressure (SCPP) as: ABP – ISP.
   1. HYPOTHESIS: ISP, ABP and SCPP will demonstrate a reproducible relationship in time both within and between patients, allowing for comment on the transmission of ABP pulsations to pulsatile spinal cord blood volume.
2. Feasibility of intra-compartmental compensatory reserve will be characterized, by evaluating the relationship between slow-wave vasogenic fluctuations in the Fast Fourier Transformed pulse amplitude of ISP (called sAMP) and ISP over time.
   1. HYPOTHESIS: It will be possible to derive a continuous metric of intra-spinal compensatory reserve (called sRAP), which represents aspects of intra-spinal compliance and will demonstrate a negative parabolic relationship to continuously measured ISP. This will mimic that seen between intra-cranial pressure and continuously measures cerebral compensatory reserve index, call RAP.
3. Spinal cord vascular reactivity will be characterized continuously using the relationship between slow-wave vasogenic fluctuations in ISP or sAMP, and either mean arterial pressure (MAP) or SCPP.
   1. HYPOTHESIS A: These spinal cord vascular reactivity indices will display characteristic parabolic relationships with MAP and SCPP, and positive correlations to progressively increasing ISP and sAMP.
   2. HYPOTHESIS B: The minimum of the parabolic relationship between spinal cord vascular reactivity metrics and MAP or SCPP will allow for continuously derived individual ‘optimal’ SCPP and ‘optimal’ MAP.

Secondary objectives of this study will be to:

1. Evaluate the safety of invasive ISP monitoring.
   1. HYPOTHESIS: Invasive intra-spinal pressure monitoring will be safe and feasible, with low complication rates.
2. Prospectively evaluate the overall relationship between spinal cord perfusion pressure and functional outcomes in patients with acute traumatic spinal cord injury.
   1. HYPOTHESIS: There will be a general positive relationship between both SCPP and individual optimal SCPP, with patient functional outcome in acute traumatic spinal cord injury.
3. Evaluate the relationship between SCPP, motor evoked potentials and functional outcomes after *incomplete* spinal cord injury.
   1. HYPOTHESIS: Time spent with SCPP near individual optimal SCPP will be directly associated with improved motor evoked potentials and functional outcomes in *incomplete* spinal cord injury.

### ***3.2* Study Outcome Measures**

*Primary Outcome Measures*

The outcome measures of interest for our primary objective and specific aims include monitoring of intraspinal pressure via a strain-gauge pressure transducing wire placed at the site of injury and the derivation of various spinal physiologic metrics (see section 4.1 in Study Design). We will focus on various signal processing techniques to derive: ISP, MAP, SCPP, sAMP, sRAP and various spinal cord vascular reactivity metrics during the post-acquisition processing phase of the study. This will allow for the evaluation of relationships between spinal physiologic metrics, as outlined in the specific aims for the primary objective.

*Secondary Outcome Measures*

Safety of intraspinal pressure monitoring will be measured based on the accuracy of placement of the probe from postoperative computed tomography (CT) scan imaging, as well as collection of adverse events including infection rates, rates of pseudomeningoceles, neurological injury, probe dislodgement, meningitis, and/or subdural hematoma at the site of probe placement.

Where functional outcomes are of interest, we will measure changes to the ASIA impairment scale, functional independence score (FIM) and Quality of Life Index for Spinal Cord Injury (QLI-SCI).

## 4. Study Design

### **4.1 Patient Population**

This study will be a single-center observational study involving patients admitted to the intensive care unit at Health Sciences Centre with acute traumatic spinal cord injury (ASIA A, B or C). All patients will undergo placement of an intraspinal pressure monitor within 24hrs of arrival. Physiological recordings will occur during the SICU stay for a maximum duration of 5 days (or until discharge from the intensive care unit).

Given that this is a feasibility and validation study, we will recruit patients over 2 years and anticipate that 20 patients will be enrolled during this time frame. Upon admission, patients will be screened for inclusion in the study based on the inclusion and exclusion criteria listed in *Section 5*. Initial demographic information, medical history, physical examination and imaging findings will be recorded using standardized data collection forms for all patients that consent to participation in the study. Patients will then be taken for surgical intervention within 24hrs of admission, and an intraspinal pressure monitor will be inserted as part of the overall surgical procedure. Initial intraspinal pressure readings will be recorded intraoperatively. Standardized data collection forms will then be used to collect pertinent data related to the surgical intervention and any intraoperative adverse events. Similarly, postoperative adverse events will be collected for the first 30 days following surgical intervention.

### **4.2 Physiologic Data Capture and Processing**

Once surgical intervention has been completed, the patient will be monitored in the intensive care unit as long as deemed necessary by care providers. During this time in SICU, intraspinal pressure and physiological parameters such as heart rate, respiratory rate, blood pressure, and mean arterial pressure will be captured at the bedside using a secure laptop with ICM+ software.

Both ABP and ISP will be obtained through invasive methods, with all signals recorded in high-frequency time series, sampled at 100 Hz or higher through and analogue to digital signal converters (DT9804 or 9T9826; Data Translations, Marlboro, MA), using ICM+ software (Cambridge Enterprise Ltd, Cambridge, UK, http://www.neurosurg.cam.ac.uk/icmplus) connected to our SICU monitors. Signals from all of the monitoring devices described below are subsequently recorded in time series using this software over the course of the recording periods described above. All physiologic signals from monitoring devices within the SICU for neurologically ill patients are currently recorded and archived as part of a separate prospective signal database study that will be ongoing at HSC (HS20840; H2017:181). Arterial blood pressure (ABP) will be obtained through either radial or femoral arterial lines connected to pressure transducers (Baxter Healthcare Corp. CardioVascular Group, Irvine, CA). ICP will be acquired via an intra-parenchymal strain gauge probe (Codman ICP MicroSensor; Codman & Shurtleff Inc., Raynham, MA).

Post-acquisition processing of the above signals will be conducted using ICM+ software. SCPP will be calculated using the formula: SCPP = MAP – ISP. Systolic ABP (ABPs) will be determined by calculating the maximum ABP over a 1.5 second window, updated every second. Similarly, diastolic ABP (ABPd) will be determined by calculating the minimum ABP over a 1.5 second window, updated every second. Pulse amplitude of ISP (sAMP) will be determined by calculating the fundamental Fourier amplitude of the ICP pulse waveforms over a 10 second window, updated every 10 seconds.

Ten-second moving averages (updated every 10 seconds to avoid data overlap) will be calculated for all recorded signals: ISP, ABP (which produced MAP), ABPs and ABPd. Ten-second moving averages will be calculated in order to focus on slow waves of parent signals, decimating the frequency to the range associated with vascular autoregulation.

Spinal vascular reactivity indices will be derived as follows: a moving Pearson correlation coefficient will be calculated between ISP and MAP using 30 consecutive 10-second windows (i.e. five minutes of data), updated every minute. This index will be referred to as the spinal pressure reactivity index (sPRx). Similarly, other vascular reactivity metrics will be derived using the correlations between slow-waves of sAMP and MAP (creating spinal pulse amplitude index; sPAx) and sAMP and SCPP (creating sRAC; R = correlation, A = pulse amplitude of ISP, C = SCPP). Finally, the spinal compensatory reserve will be assessed by deriving the spinal RAP index (sRAP), using the moving Pearson correlation between sAMP and ISP. Data for further analysis will be provided in the form of a minute by minute time trends, output into comma-separated values (CSV) datasets.

### ***4.3* Motor Evoked Potentials**

In patients undergoing evaluation for motor evoked potential monitoring, we will capture transcranial magnetic motor evoked potentials within the 24 hours pre-operatively, 24hrs post-operatively and again during the standardized functional assessments at 1, 3, 6, 9 and 12 months.

***4.4 Functional Outcome Assessments***

Each patient will then undergo standardized functional assessments at 1, 3, 6, 9, and 12 months following the acute injury. Functional assessments will include assessment of the ASIA impairment scale, functional independence score (FIM) and Quality of Life Index for Spinal Cord Injury (QLI-SCI). We may also use information captured in the Rick Hansen Spinal Cord Injury Registry, which is a national database that collects functional outcomes and adverse events on patients with traumatic spinal cord injury. This registry is currently approved by the University of Manitoba Research Ethics Board (Ethics # H2005:173(HS14502)).

**5.** Study Enrollment and Withdrawal

In this study, we aim to enroll 20 patients over the course of 2 years to evaluate the role of intraspinal pressure monitoring in the management of acute spinal cord injury. As described above, patients with acute traumatic spinal cord injury will be identified at the time of admission to the Health Sciences Center.

***5.1 Inclusion Criteria***

In order to participate in this study, patients must meet all of the following criteria:

- patients with acute traumatic spinal cord injury ASIA A, B or C
- spinal vertebral level between C0-L1
- age between 18–70yrs

### **5.2 Exclusion Criteria**

Any patients meeting the exclusion criteria below will be excluded from study participation.

- patients with cauda equina syndrome or radicular injury only
- patients presenting to HSC >48hrs from time of spinal cord injury
- patients unable to communicate in the English language
- pre-existing cognitive impairment
- penetrating spinal cord injury
- a pre-existing neurodegenerative disorder involving brain or spinal cord
- patients with concomitant injuries requiring emergent surgical intervention
- patients with injuries to the chest, abdomen, pelvis or extremities, which would impair a clinician’s ability to discern the severity of the injury
- history of coagulopathy
- pre-existing infection
- pregnancy

### **5.3 Strategies for Recruitment and Retention**

Research nurses/assistants will be employed to continually scan patient lists in the intensive care unit to identify potential candidates. Long-term retention of patients in the study will be supported via dedicated clinic follow-ups with patients. During these clinic visits, we will encourage and assist patients with the completion of standardized questionnaires. Patients will not be compensated for participation in this study.

### **5.4 Subject Withdrawal**

Study patients may withdraw voluntarily from the study, or the investigator may terminate the patient’s participation in the study if:

- a medical condition or event occurs such that ongoing participation in the study would not be in the best interest of the patient
- the subject meets an exclusion criterion (either newly developed or previously not recognized) that precludes further study participation

In situations where a patient is withdrawn from the study, complete adverse events data will be captured and stored in case of future need. We will then aim to extend the duration of the study until the study participant can be replaced with another patient meeting the inclusion criteria for the study.

### **5.5 Premature Termination or Suspension of the Study**

This study may be suspended or prematurely terminated if there is sufficient reasonable cause. Written notification, documenting the reason for study suspension or termination will be provided to the REB.

Circumstances that may warrant termination include, but are not limited to:

- determination of unexpected, significant or unacceptable risk to patients
- insufficient adherence to protocol requirements
- data that are not sufficiently complete
- determination of futility

## 6. Study Schedule

### **6.1 Schedule of Evaluations**

See Appendix 1.

### **6.2 Description of Evaluations**

**Screening Visit and Informed Consent (Day 0 to Day 1)**

All patients admitted to the surgical intensive care unit at Health Sciences Center will be screened for potential enrollment in the study. The admission lists will be reviewed to identify patients with a spinal cord injury and patients that may be potential candidates for enrollment will be consented on a screening consent form. Thereafter, hospital records will be reviewed to verify whether the patient meets any inclusion or exclusion criteria. Initial screening may also require that the patient be examined to verify cognitive status and level of consciousness.

All patients deemed eligible for participation in the study will then be consented by a study coordinator through direct patient visit in the SICU. During this visit, study participates will also be given contact information for the study coordinator and a schedule for study visits.

Patients admitted on the weekends may not be eligible for the study as study personnel and necessary equipment may not be available

**Baseline Enrollment Visit (Day 0 to Day 1)**

After obtaining consent, study personnel will:

- perform an initial clinical assessment to obtain patient’s medical history
- conduct a physical examination
- verify medications and document laboratory investigations
- record vital signs for the patient
- review clinical exam and radiographic imaging studies to derive injury classification score using TLICs or SLICs scoring systems (all patients will need CT scan of spine and MRI of spine within first 24hours of arrival to SICU)
- administer ASIA impairment scale assessment (an objective scoring system to document level of spinal cord injury)
- administer patient questionnaires including Functional Independence Score and Quality of Life Index for Spinal Cord Injury (QLI-SCI)

**Surgical Treatment Visit (Day 0 to Day 3)**

- complete anesthesia log following termination of surgery
- complete intraoperative intraspinal pressure monitoring form
- complete intraoperative neurophysiological recordings form
- document any intraoperative adverse events using the intraoperative adverse event form

**Intraspinal Pressure Monitoring Visits (POD#1 to POD#5)**

Following surgery, each patient will be visited daily to conduct the following study related procedures:

- ensure no change to clinical status of the patient that would warrant termination of that patient’s involvement in the study
- verify that the intrapsinal pressure monitor is in situ and recordings are being captured continuously
- verify continuous monitoring of physiological data
- conduct transcranial magnetic motor evoked potential testing and record findings on neurophysiological monitoring report form
- document any medical or surgical adverse events
- document any protocol violations -document laboratory investigations including blood chemistry, hematology and medication use.

**Postoperative Inpatient Visits (POD#6 to 29)**

- on POD#6, the intraspinal pressure monitor will be removed at the bedside by study personnel
- medical or surgical adverse events will be recorded

**Follow Up Visits (POD#30, 3, 6, and 12 months following surgery)**

- functional assessment questionnaires will be administered including ASIA impairment scale, Functional Independence Score (FIM) and Quality of Life Index for Spinal Cord Injury (QLI-SCI).
- medical and surgical adverse events will be recorded in preceding time window will be noted
- functional progress with rehabilitation will be documented on the Physical Therapy and Rehabiliation form
- any readmissions (where study personnel were not otherwise notified) will be recorded.

**Study Close Out (12 month follow up visit)**

Patients will be followed for one year following surgery. At the end of this time period, patients will be given a letter to document termination of their involvement in the study. Study patients will be counseled to refer any further adverse events to either the Physical Medicine and Rehabilitation Physicians or to their Spine Surgeon.

At the termination of the study, a synopsis of the study results will be mailed to the study participants along with contact information for study personnel if they have any questions or concerns.

Further management issues and follow up will be done according to the standard practices of the involved surgeon, surgical team and nursing staff.

At the conclusion of the trial, all study forms, manuals and documentation (electronic and hard copies) will be stored in a secure location at the Foothills Medical Centre (exact location to be determined). Final data checks should be completed within six weeks of study termination after which the data set will be frozen for the purposes of final analysis. Mortality and morbidity checks will also be performed at this time and will be reported to the local Research Ethics Board. Study data, forms or manuals will not be made available to outside parties until the final manuscript has been completed and unless all primary investigators agree to disseminate this information. In the case where the study is terminated to the patient safety concerns, patients will be notified immediately about the reason for cessation of the trial by study personnel.

**Any Readmissions**

Study participants and family members will be encouraged to contact study personnel to inform of any readmissions to hospital within the province. Admission records will be requested from admitting facility (if other than Health Sciences Center) to review the date of admission, cause of admission, duration of admission and diagnosis. Any medical or surgical adverse events will be noted on the appropriate Unscheduled Readmissions Form. Patients will be managed at the discretion of the treating physician.

## 7. Safety Assessments

Safety monitoring for this study will focus on unanticipated problems involving risks to participants, including unanticipated problems that meet the definition of a serious adverse event.

### **7.1 Unanticipated Problems**

Unanticipated problems involving risks to subjects or others to include, in general, any incident, experience, or outcome that meets all of the following criteria:

- unexpected in terms of nature, severity, or frequency given (a) the research procedures that are described in the protocol-related documents, such as the REB-approved research protocol and informed consent document; and (b) the characteristics of the subject population being studied;
- related or possibly related to participation in the research (possibly related means there is a reasonable possibility that the incident, experience, or outcome may have been caused by the procedures involved in the research); and
- suggests that the research places subjects or others at a greater risk of harm (including physical, psychological, economic, or social harm) than was previously known or recognized.

### **7.2 Serious Adverse Events**

A serious adverse event (SAE) is one that meets one or more of the following criteria:

- Results in death
- Is life-threatening (places the subject at immediate risk of death from the event as it occurred)
- Results in inpatient hospitalization or prolongation of existing hospitalization
- Results in a persistent or significant disability or incapacity
- Results in a congenital anomaly or birth defect

Any unanticipated or serious adverse events will be reported to the research ethics board as per the procedures documented in *Section 7.3 Reporting Procedures*.

### **7.3 Reporting Procedures**

Incidents or events that meet the criteria for unanticipated problems require the creation and completion of a serious adverse events form. Investigators will include the following information when reporting an adverse event, or any other incident, experience, or outcome as an unanticipated problem to the REB:

- appropriate identifying information for the research protocol, such as the title, investigator’s name, and the REB project number;
- a detailed description of the adverse event, incident, experience, or outcome;
- an explanation of the basis for determining that the adverse event, incident, experience, or outcome represents an unanticipated problem;
- a description of any changes to the protocol or other corrective actions that have been taken or are proposed in response to the unanticipated problem.

To satisfy the requirement for prompt reporting, unanticipated problems will be reported using the following timeline:

- Unanticipated problems that are serious adverse events will be reported to the REB within 1 week of the investigator becoming aware of the event.
- Any other unanticipated problem will be reported to the REB within 2 weeks of the investigator becoming aware of the problem.

### **7.4 Study Oversight**

The investigator will be responsible for study oversight, including monitoring safety, ensuring that the study is conducted according to the protocol and ensuring data integrity. The PI will review the data for safety concerns and data trends at regular intervals, and will promptly report to the research ethics board any Unanticipated Problem (UP), protocol deviation, or any other significant event that arises during the conduct of the study.

A safety monitoring committee will be convened to review all adverse events during the study. The safety monitoring committee will consist of the primary investigators, critical care physicians and neurosurgeons. The committee will review all adverse events for every cohort of 5 patients enrolled into the study. To ensure timely review of adverse events, the data safety monitoring committee will review adverse events within the first 30 days of the surgical and study interventions.

## 8. Statistical Considerations

### 8.1 General Statistics

Statistical analysis will be performed utilizing R statistical software (R Core Team (2018). R: A language and environment for statistical computing. R Foundation for Statistical Computing, Vienna, Austria. URL https://www.R-project.org/). Alpha for statistical significance will be set at 0.05, with normality for all continuous variables tested via Shapiro-Wilks test. Basic descriptive statistics will be performed, with comparison between groups and variables conducted via t-test, Mann-Whitney-U, chi-square, analysis of variance (ANOVA), Kruskal-Wallis, Friedman and Joncheere-Terpstra testing, where appropriate. General correlations will be described using Pearson/Spearman coefficients, where applicable.

Descriptive statistical analysis of the relationships between physiologic variables will be facilitated through various error bar plots, linear models, quadratic models (ie. For optimal SCPP determination) and locally weighted scatterplot smoothing (LOESS) techniques, depending on the physiologic relationship being assessed.

### 8.2 Time Series Techniques

All indices of cerebrovascular reactivity will be output in minute-by-minute time series format. Correlation between ISP and MAP slow-waves will be further evaluated in time series using cross correlation techniques, cross-correlation function (CCF) plots, vector autoregressive integrative moving average (VARIMA) models, impulse response function (IRF) plots and Granger causality testing. Further, using Box-Jenkins time series modelling, the autoregressive integrative moving average (ARIMA) structure of each time series will be assessed and compared. ARIMA model accuracy for each index will be confirmed using autocorrelation function (ACF), partial autocorrelation function (PACF) plot, augmented Dickey-Fuller (ADF) and Kwiatkowski–Phillips–Schmidt–Shin (KPSS) testing, and the ARIMA model superiority will be confirmed via ANOVA testing and comparing Akaike information criterion (AIC), Bayesian information criterion (BICpresence of random normally distributed residuals. VARIMA and ) and log-likelihood (LL).

### 8.3 Association with Secondary Outcomes

ISP, MAP, SCPP, sAMP, sRAP and the spinal vascular reactivity indices will be evaluated in relationship to motor evoked potential recordings, and functional outcome measures at the defined time periods of assessment. Such analysis will involve linear and logistic regression modelling techniques, evaluating the relationship between the physiologic parameters with: ASIA, FIM and QLI-SCI functional outcome metrics.

## 9. Source Documents and Access to Source Data/Documents

Study staff will maintain appropriate medical and research records for this study, in compliance with institutional requirements for the protection of confidentiality of subjects. Study staff will permit authorized representatives of regulatory agencies to examine (and when required by applicable law, to copy) research records for the purposes of quality assurance reviews, audits, and evaluation of the study safety, progress and data validity.

Data recorded during the course of the study will be documented using clinical research forms or using digital software. Hard copies of study records will be archived by the investigator in a locked room located in the office of the PI in the Section of Neurosurgery at Health Sciences Center. Access to this room is monitored by hospital security and is limited to the primary investigator and one administrative assistant. When the office is not occupied, the door will be locked at all times. Study records will be destroyed after 25 years. All patient charts will be stored according to Manitoba Health policy. Computer access is through Manitoba eHealth who maintains a firewall. Each member of the study team has their own user ID number and terminals will be logged off when not being used.

Any disposal of documents will be destroyed by placing in confidential shredding bins located on the hospital premises. Publication/presentations will be done with aggregate data and no patient identifiers will be released.

### **9.1 Data Handling and Record Keeping**

The investigators are responsible for ensuring the accuracy, completeness, legibility, and timeliness of the data reported. All source documents should be completed in a neat, legible manner to ensure accurate interpretation of data. The investigators will maintain adequate case histories of study participants, including accurate case report forms (CRFs), and source documentation.

### **9.2 Data Management Responsibilities**

Data collection and accurate documentation are the responsibility of the study staff under the supervision of the investigator. All source documents and laboratory reports must be reviewed by the study team and data entry staff, who will ensure that they are accurate and complete. Unanticipated problems must be reviewed by the investigator or designee.

## 10 Ethics And Protection of Human Subjects

### **10.1 Research Ethics Board Submissions**

The protocol, informed consent form(s), recruitment materials and all participant materials will be submitted to the REB for review and approval. Approval of both the protocol and the consent form must be obtained before any participant is enrolled. Any amendment to the protocol will require review and approval by the REB before the changes are implemented in the study.

### **10.2 Informed Consent**

Informed consent will be obtained prior to the individual agreeing to participate in the study and will continue throughout study participation. Extensive discussion of risks and possible benefits of study participation will be provided to participants and their families. A consent form describing in detail the study procedures and risks will be given to the participant. Consent forms will be REB-approved, and the participant is required to read and review the document or have the document read to him or her. The investigator or designee will explain the research study to the participant and answer any questions that may arise. The participant will sign the informed consent document prior to any study-related assessments or procedures. Participants will be given the opportunity to discuss the study with their surrogates or think about it prior to agreeing to participate. They may withdraw consent at any time throughout the course of the study. A copy of the signed informed consent document will be given to participants for their records. The rights and welfare of the participants will be protected by emphasizing to them that the quality of their clinical care will not be adversely affected if they decline to participate in this study.

The consent process will be documented in the clinical or research record. If the patient is under the clinical care of the principal investigator for the study, then another member of the research team will become involved to obtain consent from the patient.

### **10.3 Patient Confidentiality**

Participant confidentiality is strictly held in trust by the investigators, study staff, and the sponsor(s) and their agents. This confidentiality is extended to cover testing of biological samples and genetic tests in addition to any study information relating to participants.

The study protocol, documentation, data, and all other information generated will be held in strict confidence. No information concerning the study or the data will be released to any unauthorized third party without prior written approval of the REB.

## 11. References

1. Cripps RA, Lee BB, Wing P, Weerts E, Mackay J, Brown D. A global map for traumatic spinal cord injury epidemiology: Towards a living data repository for injury prevention. *Spinal Cord*. 2011;49(4):493-501. doi:10.1038/sc.2010.146

2. Fehlings MG, Vaccaro A, Wilson JR, et al. Early versus Delayed Decompression for Traumatic Cervical Spinal Cord Injury: Results of the Surgical Timing in Acute Spinal Cord Injury Study (STASCIS). Di Giovanni S, ed. *PLoS ONE*. 2012;7(2):e32037. doi:10.1371/journal.pone.0032037

3. Tator CH. Experimental and Clinical Studies of the Pathophysiology and Management of Acute Spinal Cord Injury. *J Spinal Cord Med*. 1996;19(4):206-214. doi:10.1080/10790268.1996.11719436

4. Becker D, Sadowsky CL, McDonald JW. Restoring Function After Spinal Cord Injury. *The Neurologist*. 2003;9(1):1-15. doi:10.1097/01.nrl.0000038587.58012.05

5. Tykocki T, Poniatowski Ł, Czyż M, Koziara M, Wynne-Jones G. Intraspinal Pressure Monitoring and Extensive Duroplasty in the Acute Phase of Traumatic Spinal Cord Injury: A Systematic Review. *World Neurosurg*. 2017;105:145-152. doi:10.1016/j.wneu.2017.05.138

6. Sabit B, Zeiler FA, Berrington N. The Impact of Mean Arterial Pressure on Functional Outcome Post Trauma-Related Acute Spinal Cord Injury: A Scoping Systematic Review of the Human Literature. *J Intensive Care Med*. 2018;33(1):3-15. doi:10.1177/0885066616672643

7. Ryken TC, Hurlbert RJ, Hadley MN, et al. The Acute Cardiopulmonary Management of Patients With Cervical Spinal Cord Injuries: *Neurosurgery*. 2013;72:84-92. doi:10.1227/NEU.0b013e318276ee16

8. Werndle MC, Saadoun S, Phang I, et al. Monitoring of Spinal Cord Perfusion Pressure in Acute Spinal Cord Injury: Initial Findings of the Injured Spinal Cord Pressure Evaluation Study*. *Crit Care Med*. 2014;42(3):646-655. doi:10.1097/CCM.0000000000000028

## Appendix 1 – Study Schedule

| Assessment | Screening Visit | Baseline Enrollment Visit | Surgical Treatment Visit | Intraspinal Pressure Monitoring Visit 1 | Intraspinal Pressure Monitoring Visit 2 | Intraspinal Pressure Monitoring Visit 3 | Intraspinal Pressure Monitoring Visit 4 | Intraspinal Pressure Monitoring Visit 5 | Postoperative In-hospital visits | Any Readmission | Follow Up Visit 1 (1 month after surgery | Follow up Visit 2 (3 months after surgery) | Follow Up Visit 3 (6 months After surgery) | Follow Up Visit 4 (9 months after surgery) | Follow Up Visit 5 (12 months after surgery) |
| --- | --- | --- | --- | --- | --- | --- | --- | --- | --- | --- | --- | --- | --- | --- | --- |
| Time Frame | Day 0 to 1 | Day 0 to 1 | Day 0 to 3 | POD#1 | POD #2 | POD #3 | POD #4 | POD#5 | POD#6-29 | Any Readmission | POD#30 | 3 months following surgery +/-14days) | 6 months following surgery +/-14days) | 9 months following surgery +/-14days) | 12 months following surgery +/-14days) |
| Screening Log |  |  |  |  |  |  |  |  |  |  |  |  |  |  |  |
| Confirmationation of Inclusion/Exclusion Criteria |  |  |  |  |  |  |  |  |  |  |  |  |  |  |  |
| Informed Consent |  |  |  |  |  |  |  |  |  |  |  |  |  |  |  |
| Initial Clinical Assessment |  |  |  |  |  |  |  |  |  |  |  |  |  |  |  |
| Medical History |  |  |  |  |  |  |  |  |  |  |  |  |  |  |  |
| Physical Examination |  |  |  |  |  |  |  |  |  |  |  |  |  |  |  |

| Assessment | Screening Visit | Baseline Enrollment Visit | Surgical Treatment Visit | Intraspinal Pressure Monitoring Visit 1 | Intraspinal Pressure Monitoring Visit 2 | Intraspinal Pressure Monitoring Visit 3 | Intraspinal Pressure Monitoring Visit 4 | Intraspinal Pressure Monitoring Visit 5 | Postoperative In-hospital visits | Any Readmission | Follow Up Visit 1 (1 month after surgery | Follow up Visit 2 (3 months after surgery) | Follow Up Visit 3 (6 months After surgery) | Follow Up Visit 4 (9 months after surgery) | Follow Up Visit 5 (12 months after surgery) |
| --- | --- | --- | --- | --- | --- | --- | --- | --- | --- | --- | --- | --- | --- | --- | --- |
| Time Frame | Day 0 to 1 | Day 0 to 1 | Day 0 to 3 | POD#1 | POD #2 | POD #3 | POD #4 | POD#5 | POD#6-29 | Any Readmission | POD#30 | 3 months following surgery +/-14days) | 6 months following surgery +/-14days) | 9 months following surgery +/-14days) | 12 months following surgery +/-14days) |
| Medications |  |  |  |  |  |  |  |  |  |  |  |  |  |  |  |
| Laboratory Investigations (Hematology and chemistry) |  |  |  |  |  |  |  |  |  |  |  |  |  |  |  |
| Vital Signs |  |  |  |  |  |  |  |  |  |  |  |  |  |  |  |
| Clinical and Radiographic Injury Classification |  |  |  |  |  |  |  |  |  |  |  |  |  |  |  |
| ASIA Impairment Scale |  |  |  |  |  |  |  |  |  |  |  |  |  |  |  |
| Functional Independence Measure |  |  |  |  |  |  |  |  |  |  |  |  |  |  |  |
| Assessment | Screening Visit | Baseline Enrollment Visit | Surgical Treatment Visit | Intraspinal Pressure Monitoring Visit 1 | Intraspinal Pressure Monitoring Visit 2 | Intraspinal Pressure Monitoring Visit 3 | Intraspinal Pressure Monitoring Visit 4 | Intraspinal Pressure Monitoring Visit 5 | Postoperative In-hospital visits | Any Readmission | Follow Up Visit 1 (1 month after surgery | Follow up Visit 2 (3 months after surgery) | Follow Up Visit 3 (6 months After surgery) | Follow Up Visit 4 (9 months after surgery) | Follow Up Visit 5 (12 months after surgery) |
| Time Frame | Day 0 to 1 | Day 0 to 1 | Day 0 to 3 | POD#1 | POD #2 | POD #3 | POD #4 | POD#5 | POD#6-29 | Any Readmission | POD#30 | 3 months following surgery +/-14days) | 6 months following surgery +/-14days) | 9 months following surgery +/-14days) | 12 months following surgery +/-14days) |
| Quality of Life Index for Spinal Cord Injury |  |  |  |  |  |  |  |  |  |  |  |  |  |  |  |
| Anesthesia Log |  |  |  |  |  |  |  |  |  |  |  |  |  |  |  |
| Intraoperative Intraspinal Pressure Monitoring Form |  |  |  |  |  |  |  |  |  |  |  |  |  |  |  |
| Intraoperative neurophysiological record form |  |  |  |  |  |  |  |  |  |  |  |  |  |  |  |
| Intraoperative adverse event form |  |  |  |  |  |  |  |  |  |  |  |  |  |  |  |
| ICU Physiological Recordings |  |  |  |  |  |  |  |  |  |  |  |  |  |  |  |
| Assessment | Screening Visit | Baseline Enrollment Visit | Surgical Treatment Visit | Intraspinal Pressure Monitoring Visit 1 | Intraspinal Pressure Monitoring Visit 2 | Intraspinal Pressure Monitoring Visit 3 | Intraspinal Pressure Monitoring Visit 4 | Intraspinal Pressure Monitoring Visit 5 | Postoperative In-hospital visits | Any Readmission | Follow Up Visit 1 (1 month after surgery | Follow up Visit 2 (3 months after surgery) | Follow Up Visit 3 (6 months After surgery) | Follow Up Visit 4 (9 months after surgery) | Follow Up Visit 5 (12 months after surgery) |
| Time Frame | Day 0 to 1 | Day 0 to 1 | Day 0 to 3 | POD#1 | POD #2 | POD #3 | POD #4 | POD#5 | POD#6-29 | Any Readmission | POD#30 | 3 months following surgery +/-14days) | 6 months following surgery +/-14days) | 9 months following surgery +/-14days) | 12 months following surgery +/-14days) |
| ICU Intraspinal Pressure Recordings |  |  |  |  |  |  |  |  |  |  |  |  |  |  |  |
| Neurophysiological Monitoring Report |  |  |  |  |  |  |  |  |  |  |  |  |  |  |  |
| Spinal Cord Injury – Secondary Complications Form |  |  |  |  |  |  |  |  |  |  |  |  |  |  |  |
| Unscheduled Readmissions Form |  |  |  |  |  |  |  |  |  |  |  |  |  |  |  |
| Postoperative Medial Adverse Events |  |  |  |  |  |  |  |  |  |  |  |  |  |  |  |

| Assessment | Screening Visit | Baseline Enrollment Visit | Surgical Treatment Visit | Intraspinal Pressure Monitoring Visit 1 | Intraspinal Pressure Monitoring Visit 2 | Intraspinal Pressure Monitoring Visit 3 | Intraspinal Pressure Monitoring Visit 4 | Intraspinal Pressure Monitoring Visit 5 | Postoperative In-hospital visits | Any Readmission | Follow Up Visit 1 (1 month after surgery | Follow up Visit 2 (3 months after surgery) | Follow Up Visit 3 (6 months After surgery) | Follow Up Visit 4 (9 months after surgery) | Follow Up Visit 5 (12 months after surgery) |
| --- | --- | --- | --- | --- | --- | --- | --- | --- | --- | --- | --- | --- | --- | --- | --- |
| Time Frame | Day 0 to 1 | Day 0 to 1 | Day 0 to 3 | POD#1 | POD #2 | POD #3 | POD #4 | POD#5 | POD#6-29 | Any Readmission | POD#30 | 3 months following surgery +/-14days) | 6 months following surgery +/-14days) | 9 months following surgery +/-14days) | 12 months following surgery +/-14days) |
| Postoperative Surgical Adverse Events Report |  |  |  |  |  |  |  |  |  |  |  |  |  |  |  |
| Protocol Violation Report |  |  |  |  |  |  |  |  |  |  |  |  |  |  |  |
| Physical Therapy and Rehabilitation Form |  |  |  |  |  |  |  |  |  |  |  |  |  |  |  |
| Close Out Letter to Patient |  |  |  |  |  |  |  |  |  |  |  |  |  |  |  |

APPENDIX 2 – STUDY VERSION HISTORY

| VERSION | PURPOSE OR CHANGE | DATE DOCUMENT CHANGED | AUTHOR |
| --- | --- | --- | --- |
| 3.0 | Change inclusion and exclusion criteria to align with CAMPER trial. Notes to add a data safety monitoring committee were also added. | November 09, 2021 | Perry Dhaliwal |
